# Reducing variability among treatment machines using knowledge‐based planning for head and neck, pancreatic, and rectal cancer

**DOI:** 10.1002/acm2.13316

**Published:** 2021-06-20

**Authors:** Hideaki Hirashima, Mitsuhiro Nakamura, Nobutaka Mukumoto, Ryo Ashida, Kota Fujii, Kiyonao Nakamura, Aya Nakajima, Katsuyuki Sakanaka, Michio Yoshimura, Takashi Mizowaki

**Affiliations:** ^1^ Department of Radiation Oncology and Image‐applied Therapy Graduate School of Medicine Kyoto University Kyoto Japan; ^2^ Division of Medical Physics, Department of Information Technology and Medical Engineering Faculty of Human Health Science Graduate School of Medicine Kyoto University Kyoto Japan

**Keywords:** different treatment machine, disease site, energy, knowledge‐based planning, multileaf collimator type

## Abstract

**Purpose:**

This study aimed to assess dosimetric indices of RapidPlan model‐based plans for different energies (6, 8, 10, and 15 MV; 6‐ and 10‐MV flattening filter‐free), multileaf collimator (MLC) types (Millennium 120, High Definition 120, dual‐layer MLC), and disease sites (head and neck, pancreatic, and rectal cancer) and compare these parameters with those of clinical plans.

**Methods:**

RapidPlan models in the Eclipse version 15.6 were used with the data of 28, 42, and 20 patients with head and neck, pancreatic, and rectal cancer, respectively. RapidPlan models of head and neck, pancreatic, and rectal cancer were created for TrueBeam STx (High Definition 120) with 6 MV, TrueBeam STx with 10‐MV flattening filter‐free, and Clinac iX (Millennium 120) with 15 MV, respectively. The models were used to create volumetric‐modulated arc therapy plans for a 10‐patient test dataset using all energy and MLC types at all disease sites. The Holm test was used to compare multiple dosimetric indices in different treatment machines and energy types.

**Results:**

The dosimetric indices for planning target volume and organs at risk in RapidPlan model‐based plans were comparable to those in the clinical plan. Furthermore, no dose difference was observed among the RapidPlan models. The variability among RapidPlan models was consistent regardless of the treatment machines, MLC types, and energy.

**Conclusions:**

Dosimetric indices of RapidPlan model‐based plans appear to be comparable to the ones based on clinical plans regardless of energies, MLC types, and disease sites. The results suggest that the RapidPlan model can generate treatment plans independent of the type of treatment machine.

## 1 INTRODUCTION

To achieve clinical goals using volumetric‐modulated arc therapy (VMAT), it is important to understand optimization methods and patient conditions. Thus, plan quality and optimization time of VMAT is dependent on the planners' knowledge and experience. Knowledge‐based VMAT planning was developed to minimize interplanner variability and improve plan quality.^1^ Methods for knowledge‐based planning can be further divided into two major categories: atlas‐based methods and statistical modeling (including machine learning methods).^1^ One of the statistical modeling methods is RapidPlan (Varian Medical Systems, Palo Alto, CA), a commercial knowledge‐based planning solution derived from earlier work, which uses a model based on a library of previous plans.^2^, ^3^, ^4^, ^5^, ^6^, ^7^, ^8^, ^9^, ^10^, ^11^ The model can be used to predict a range of achievable organ at risk (OAR) dose‐volume histograms (DVHs) for new patients. It is possible to share models among different clinical institutes in a cooperative framework.^12^, ^13^, ^14^, ^15^, ^16^

Although knowledge‐based planning using RapidPlan ensures efficiency in clinical practice, the model parameters in RapidPlan affect the quality of the predicted DVHs.^17^ The statistical information in RapidPlan models varies according to the plan and treatment machine parameters due to the geometry‐based expected dose (GED) calculation step.^18^, ^19^, ^20^, ^21^ Thus, it is important to understand model characteristics in RapidPlan in more detail. GED‐related factors may include the relationship between the geometric and dosimetric features of the planning target volume (PTV) and OARs of the library plans. Parameters of the treatment machine, such as the energy and multileaf collimator (MLC) type, can additionally affect the GED calculation step. Huang et al. demonstrated that a RapidPlan model configured for flattened beams cannot optimize unflattened beams before adjusting the target objectives.^21^ They reported the effect of different energy types on RapidPlan performance for pelvic regions^21^; however, the report was limited, and the other disease sites and machine parameters were not mentioned. Thus, the effect of the type of treatment machine in the model library on RapidPlan performance remains unknown. In addition, it remains unclear how the energy and MLC width of RapidPlan models depend on the treated disease.

This study aimed to assess dosimetric indices under different energy and MLC‐type conditions for each disease site to compare RapidPlan models with the clinical plan. We chose several energy parameters [6, 8, 10, and 15 MV; 6‐ and 10‐MV flattening filter‐free (FFF)) and MLC types {5 mm [Millennium 120], 2.5 mm [high definition (HD) 120], and dual‐layer MLC} for each disease site [head and neck cancer (HNC), pancreatic cancer (PK)], and rectal cancer (RC)].

## 2 METHODS

### 2.1 Patient enrollment

We enrolled 38, 52, and 30 consecutive patients with oro‐ or hypo‐pharynx HNC, PK, and RC, respectively, who underwent VMAT between January 2015 and November 2019. Only patients who underwent VMAT using the same treatment machine for each disease site were included. This study was performed according to the Declaration of Helsinki and was approved by the institutional review board (approval number R1446).

### 2.2 Contouring and treatment plan

All critical structures, such as OARs, were contoured by radiation oncologists and medical physicists. Target volumes were contoured by radiation oncologists. In addition, all plans were optimized by several expert radiation oncologists and medical physicists who were responsible for the protocol in clinical practice at the time of model generation.^22^, ^23^, ^24^ The radiation dose calculation algorithm used for Eclipse was Acuros XB (dose‐to‐medium) with heterogeneity correction. The calculation grid size was 2.5 mm.

Radiotherapy treatment in simultaneous integrated boost VMAT of HNC patients was set to 70 Gy in 35 fractions using TrueBeam STx (Varian Medical Systems) with 6 MV. The gross tumor volume (GTV) was defined as the gross extent of tumor evident in computed tomography (CT) images, including both the primary tumor and gross regional lymph nodes. The clinical target volume (CTV) was defined as the GTV plus a margin allowing for potential microscopic tumor extension and encompassing the adjacent regional lymph nodes. The PTV was the CTV plus a 5‐mm‐wide margin to allow for uncertainties in radiation delivery and the internal and set‐up margins. The GTV, CTV, and PTV were defined according to the contouring policy described in a previous report.^22^ The PTV70 volume included the primary tumor and lymph node metastases, whereas PTV63 and PTV56 volumes included high‐risk and low‐risk lymph nodes, respectively. The spinal cord and the left and right parotid glands were evaluated as OARs. The prescription dose was specified as D_50%_ (the dose that covers 50% of the structure) to PTV. The dose constraints are shown in the supporting information Table S1.

In PK patients, treatment prescription was set to 45 Gy in 15 fractions using TrueBeam STx with 10‐MV FFF. The target delineation, including GTV, CTV, and PTV, is described in the study by Goto et al.^23^ The prescription dose was specified as D_95%_ to PTV. The spinal cord, stomach, and duodenum were defined as OARs. The dose constraints for OARs based on a previous institutional trial are shown in supporting information Table S2.

Furthermore, RC patients for whom the 15 MV Clinac21 iX (Varian Medical Systems) was used were enrolled. CT scans were obtained in the supine (*n* = 7) and prone (*n* = 23) positions. A mixture of prone and supine cases was used due to the use of belly board since 2017. The treatment prescription was set to 45 Gy in 25 fractions, specified as D_50%_ to PTV. The CTV for the primary tumor and metastatic lymph nodes was created by adding 5 mm to the primary tumor.^24^ The PTV was based on the CTV expanded by 5 mm. The small and large bowels were evaluated as OARs. The dose constraints are shown in supporting information Table S3.

### 2.3 RapidPlan model creation

**Fig. 1:**
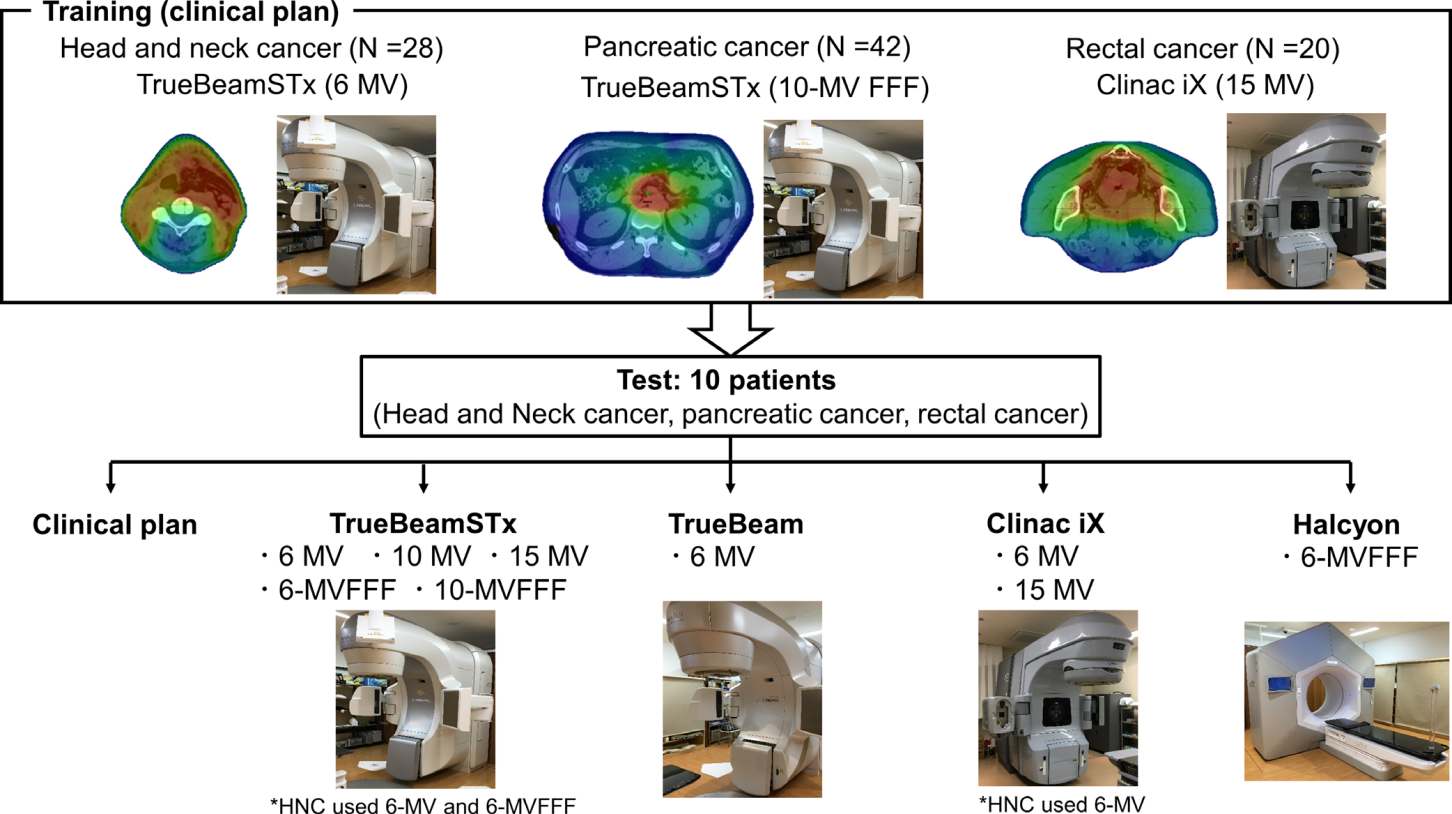
Overall scheme of this study.

The overall study scheme is shown in Fig. 1. The dataset was divided into training and test datasets. To create a RapidPlan model in the Eclipse version 15.6 (Varian Medical Systems), 28 patients with HNC, 42 patients with PK, and 20 patients with RC were used for training the model. Briefly, the RapidPlan algorithm comprises four main subsystems: (a) data extraction phase, (b) model training phase based on the GED, (c) regression analysis of all modeled structures, and (d) generation of DVH estimates (upper and lower bound) and objectives. Thereafter, the statistical tool “Model Analytics” (Varian Medical Systems, Palo Alto, CA) was employed to evaluate (and possibly exclude) potential outliers. A large number of dosimetric outliers worsened the quality of the resulting plan; therefore, all outliers were excluded in the model to eliminate any possible effect.^18^ The position and priority of DVH constraints using the RapidPlan‐generated DVHs were fine‐tuned through several trial‐and‐error attempts in five sample patients using a closed‐loop process.^6^ These constraints could be set as follows: lower, upper, generalized equivalent uniform dose (gEUD), mean dose, and line dose. Fixed priorities and ones provided by RapidPlan were used for upper and lower objectives and gEUD objectives in the target structures. Furthermore, upper gEUD, upper point objective, the line objective, and priority provided by the RapidPlan were used for the OARs. The line objective was placed just below the inferior boundary of the DVH estimated range. The values of fixed priorities were determined from the optimization results of the validation set, which were tuned to achieve our institution's acceptance criteria. The prescription setting was the same as for the clinical treatment plan. The RapidPlan templates for planning optimization of all disease sites are shown in supporting information Tables [Link], [Link], [Link].

### 2.4 Dosimetric assessment

The models were used to create VMAT plans for a 10‐patient test dataset using an open‐loop process.^6^ The treatment machine information is shown in supporting information Table S7. The RapidPlan model was calculated in this test dataset for all energy and MLC types at all disease sites. In the HNC group, the 6‐MV and 6‐MV FFF energy types were only used because other high‐energy parameters are inadequate in clinical practice due to the lower skin dose. All RapidPlan models were compared in terms of PTV and OAR parameters against original clinical plans. Selected relevant dose statistics and dose‐volume parameters were considered. Concerning PTVs, D_98%_ and D_2%_ were considered. Regarding OARs, the following parameters were assessed: D_max_ in the spinal cord and D_mean_ in the parotids for HNC, D_max_ in the spinal cord, as well as V_36Gy_ and V_39Gy_ in the stomach and duodenum for PK, and V_15Gy_ in the large and small bowels for RC.

### 2.5 Statistical analysis

Volume differences between training and test datasets were analyzed using the Student *t*‐test, Welch's *t*‐test, or the Wilcoxon rank‐sum test, taking into account the results of the normality test and equal variance test. The Holm test was used for comparisons of multiple dosimetric indices in different treatment machines and energy types to assess the statistically significant differences. A statistically significant difference was defined as *P* < 0.05.

## 3 RESULTS

The heterogeneity of the test dataset was appraised in terms of the variability of PTV and main OAR volumes for each disease site. Supporting information Table S8 shows the comparison between the training and test datasets for PTV and OAR volumes. Only the difference in duodenum volumes between the training and test datasets was statistically significant (*P* = 0.02).

**Fig. 2:**
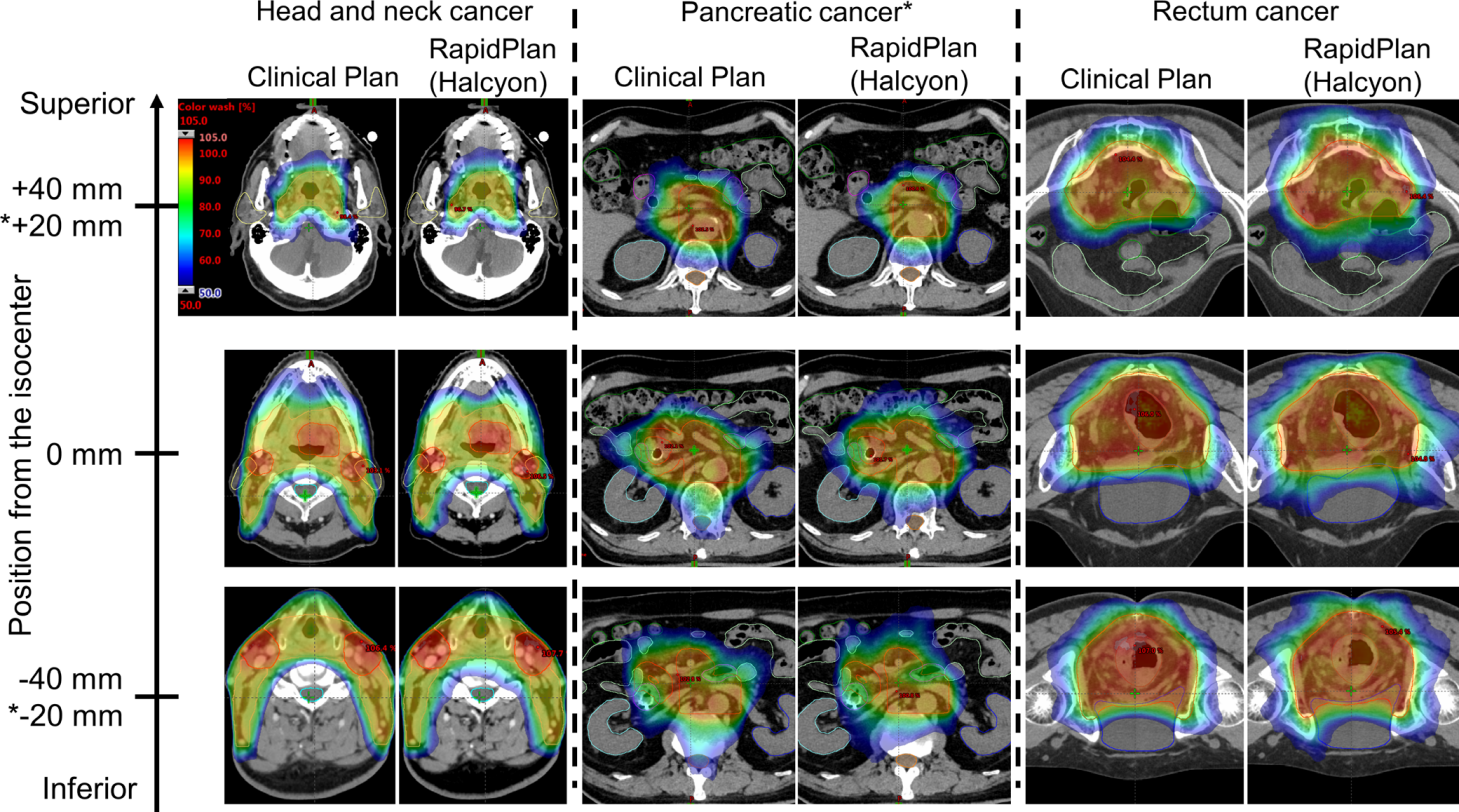
Dose distribution of both clinical plan and RapidPlan model‐based plan (e.g., Halcyon) in head and neck cancer, pancreatic cancer, and rectal cancer.

Example dose distribution of both clinical plan and RapidPlan model‐based plan in HNC, PC, and RC is shown in Fig. 2. The mean ± standard deviation (SD) of the dosimetric indices in the HNC group is shown in Table 1. The difference in D_98%_ for PTV70 between the clinical plan and Halcyon showed an improvement of 1.9% (*P *= 0.04). For all other indices, statistically significant differences in the dosimetric indices were not observed when comparing the clinical plan with the RapidPlan models using other treatment machines in the test dataset. Target and OAR dose distribution created by RapidPlan models under the condition of different energy parameters and MLC types were comparable to those of the clinical plan. There was also no difference between the clinical plan with HD MLC and RapidPlan model‐based plan with Millennium or dual‐layer MLC. Moreover, RapidPlan models using all combinations of treatment machines and energy types were also compared; thus, no differences between the compared dosimetric indices were observed.

**Table 1:** The mean ± standard deviation of dosimetric indices for head and neck cancer.

Organ	Dosimetric indices	Clinical plan	TrueBeamSTx 6X	TrueBeamSTx 6X‐FFF	TrueBeam 6X	Clinac iX 6X	Halcyon 6X‐FFF
PTV70	D_98%_ (Gy)	**65.2 ± 1.0**	66.3 ± 1.3	66.4 ± 1.2	66.2 ± 1.3	66.2 ± 1.3	**66.5 ± 0.5**
	D_2%_ (Gy)	73.1 ± 0.8	73.1 ± 0.5	73.4 ± 0.3	73.2 ± 0.4	73.2 ± 0.4	73.3 ± 0.4
PTV63	D_98%_ (Gy)	59.3 ± 0.9	58.9 ± 1.5	58.9 ± 1.6	58.7 ± 1.5	58.6 ± 1.8	59.6 ± 0.5
	D_2%_ (Gy)	67.6 ± 0.7	67.7 ± 0.6	67.9 ± 0.5	67.8 ± 0.6	67.8 ± 0.7	67.7 ± 0.5
PTV56	D_98%_ (Gy)	59.3 ± 0.9	58.9 ± 1.5	58.9 ± 1.6	58.7 ± 1.5	58.6 ± 1.8	59.6 ± 0.5
	D_2%_ (Gy)	61.7 ± 2.1	60.4 ± 1.9	60.8 ± 1.8	60.7 ± 1.8	60.6 ± 1.9	60.5 ± 1.7
Spinal cord	D_max_ (Gy)	41.2 ± 3.5	40.3 ± 4.9	40.3 ± 5.2	41.0 ± 6.6	41.2 ± 5.8	38.0 ± 4.5
Left parotid	D_mean_ (Gy)	28.8 ± 4.4	28.9 ± 5.3	28.8 ± 5.4	29.2 ± 5.4	29.0 ± 5.4	29.9 ± 6.3
Right parotid	D_mean_ (Gy)	35.7 ± 9.4	31.9 ± 6.8	31.8 ± 6.9	32.2 ± 6.9	31.9 ± 6.9	32.9 ± 7.9

Note

Values in bold represent a statistically significant difference clinical plan vs Halcyon, p = 0.04).

Abbreviations: PTV, planning target volume; D_xx%_, dose covering xx% volume of region of structure; FFF, flattening filter free.

For PK, the mean ± SD of the dosimetric indices is summarized in Table 2. The mean values for V_39Gy_ at the duodenum for the clinical plan, TrueBeam STx 6‐MV FFF, and Halcyon were 0.46, 0.03, and 0.02 cc, respectively. The difference in V_39Gy_ at the duodenum indicated a significant improvement in TrueBeam STx 6‐MV FFF and Halcyon compared with that in the clinical plan (clinical plan vs. TrueBeam STx 6‐MV FFF, *P* = 0.005; clinical plan vs. Halcyon, *P* = 0.01). All OAR dosimetric indices were reduced by RapidPlan models compared with the clinical plan. An effect of energy and MLC types on dosimetric indices was not observed among RapidPlan models. V_36Gy_ and V_39Gy_ were evaluated at the border of the dose distribution between PTV and OARs, such as the stomach and duodenum. These statistical differences were not observed between the clinical plan and RapidPlan model‐based plans due to the difference in MLC and beam energies types. Of note, the variability among RapidPlan model‐based plans was consistent regardless of the treatment machines, MLC types, and energy.

**Table 2:** The mean ± standard deviation of dosimetric indices for pancreatic cancer.

Organ	Dosimetric indices	Clinical plan	TrueBeamSTx 6X	TrueBeamSTx 6X‐FFF	TrueBeamSTx 10X	TrueBeamSTx 10X‐FFF	TrueBeamSTx 15X	TrueBeam 6X	Clinac iX 6X	Clinac iX 15X	Halcyon 6X‐FFF
PTV	D_98%_ (Gy)	36.5 ± 0.4	36.2 ± 0.2	36.1 ± 0.3	36.1 ± 0.3	36.1 ± 0.4	36.1 ± 0.3	36.1 ± 0.3	36.2 ± 0.3	36.1 ± 0.3	36.1 ± 0.1
	D_2%_ (Gy)	45.4 ± 0.2	44.8 ± 0.6	44.8 ± 0.5	44.9 ± 0.6	45.0 ± 0.6	44.9 ± 0.6	45.0 ± 0.6	45.0 ± 0.5	45.1 ± 0.5	44.6 ± 0.5
Spinal Cord	D_max_ (Gy)	23.8 ± 3.7	24.4 ± 2.9	24.0 ± 2.2	24.7 ± 1.8	23.7 ± 2.1	23.9 ± 2.6	25.4 ± 3.1	25.2 ± 1.9	25.5 ± 2.2	23.5 ± 2.3
Stomach	V_36Gy_ (cc)	3.5 ± 1.8	2.5 ± 1.5	2.7 ± 1.7	2.0 ± 1.4	2.3 ± 1.8	2.1 ± 1.5	2.4 ± 1.5	2.6 ± 1.6	2.2 ± 1.4	2.3 ± 1.5
Stomach	V_39Gy_ (cc)	0.3 ± 0.3	0.0 ± 0.1	0.1 ± 0.1	0.1 ± 0.2	0.1 ± 0.1	0.1 ± 0.2	0.1 ± 0.1	0.1 ± 0.2	0.1 ± 0.2	0.0 ± 0.1
Duodenum	V_36Gy_ (cc)	4.9 ± 4.4	4.3 ± 5.2	4.0 ± 4.4	4.1 ± 4.9	4.4 ± 5.4	4.0 ± 4.7	4.2 ± 4.8	4.2 ± 4.7	4.0 ± 4.6	3.7 ± 4.5
Duodenum	V_39Gy_ (cc)	**0.5** ± **0.3**	0.1 ± 0.4	**0.0** ± **0.1**	0.2 ± 0.5	0.2 ± 0.5	0.1 ± 0.4	0.2 ± 0.6	0.2 ± 0.5	0.3 ± 0.7	**0.0 ± 0.0**

Note

Values in bold represent a statistically significant difference (clinical plan vs. TrueBeam STx 6‐MV FFF, *P* = 0.005; clinical plan vs. Halcyon, *P* = 0.01).

Abbreviations: PTV, planning target volume; FFF, flattening filter free; D_xx%_, dose covering xx% volume of region of structure; V_yyGy_, volume receiving yy Gy.

For RC, the mean ± SD of the dosimetric indices is shown in Table 3. RapidPlan model was able to generate models comparable to those for the clinical plan when assessing the thickest region in the patient. Furthermore, differences in the treatment machine parameters (MLC type and energy) in RapidPlan model‐based plans were not statistically significant; therefore, the RapidPlan model‐based plans created with equivalent dosimetric indices for the thickest body region regardless of the MLC type and energy.

**Table 3:** The mean ± standard deviation of dosimetric indices for rectal cancer.

Organ	Dosimetric indices	Clinical plan	TrueBeamSTx 6X	TrueBeamSTx 6X‐FFF	TrueBeamSTx 10X	TrueBeamSTx 10X‐FFF	TrueBeamSTx 15X	TrueBeam 6X	Clinac iX 6X	Clinac iX 15X	Halcyon 6X‐FFF
PTV	D_98%_ (Gy)	41.2 ± 0.9	41.6 ± 0.9	41.7 ± 0.7	41.6 ± 0.9	41.7 ± 0.5	41.6 ± 0.7	41.7 ± 0.8	41.7 ± 0.7	41.6 ± 0.8	41.6 ± 1.1
	D_2%_ (Gy)	47.0 ± 0.3	46.5 ± 0.2	46.7 ± 0.3	46.5 ± 0.3	46.6 ± 0.3	46.5 ± 0.2	46.5 ± 0.2	46.5 ± 0.2	46.6 ± 0.2	46.7 ± 0.1
Large Bowel	V_15Gy_ (cc)	82.0 ± 62.8	110.8 ± 119.0	105.9 ± 103.2	104.7 ± 103.4	101.5 ± 93.2	100.3 ± 93.0	105.7 ± 100.9	108.2 ± 106.3	105.2 ± 100.9	107.2 ± 108.7
Small Bowel	V_15Gy_ (cc)	153.5 ± 121.0	197.9 ± 132.7	200.8 ± 133.2	192.8 ± 126.3	191.7 ± 119.8	191.3 ± 122.6	195.3 ± 124.4	202.6 ± 132.4	194.4 ± 125.9	198.6 ± 132.8

Abbreviations: PTV, planning target volume; FFF, flattening filter free; D_xx%_, dose covering xx% volume of region of structure; V_yyGy_, volume receiving yy Gy.

**Fig. 3:**
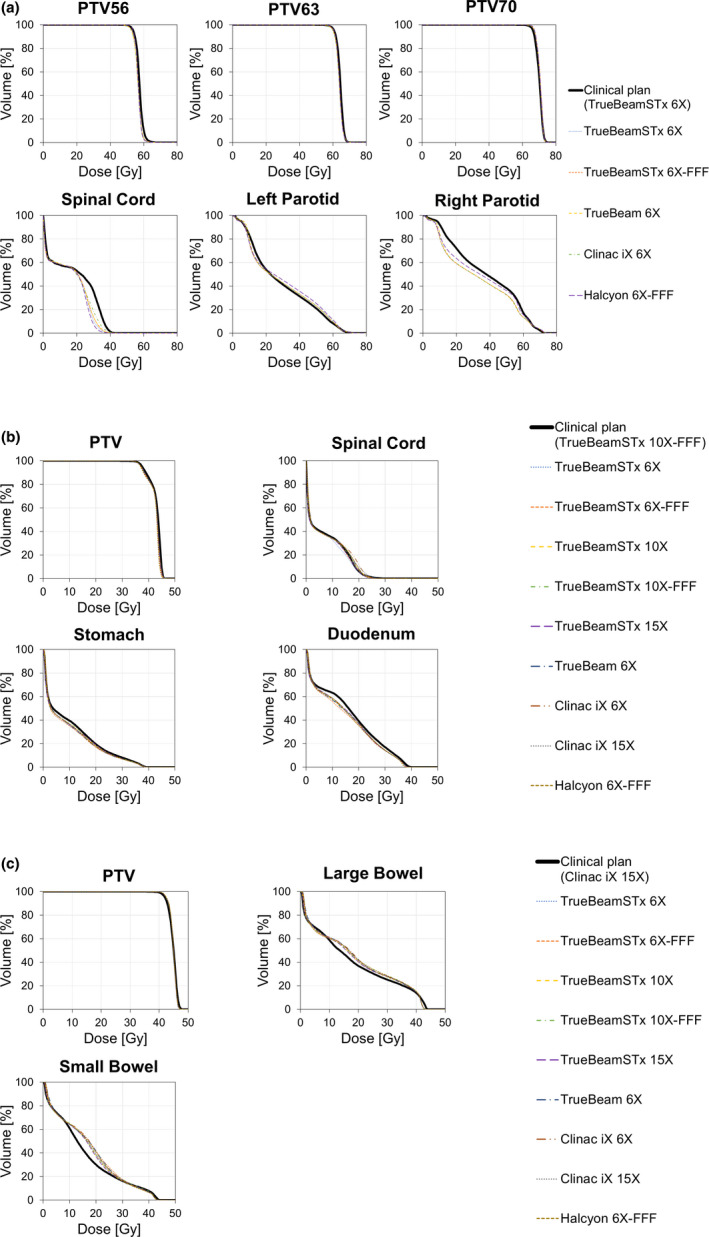
Average predicted dose‐volume histograms in the treatment of (a) head and neck cancer, (b) pancreatic cancer, and (c) rectal cancer.

The average DVHs of the PTVs and the relevant OARs for the three disease sites and for the various RapidPlan models are displayed in Fig. 3. The RapidPlan data demonstrate that qualitatively all approaches resulted in clinically equivalent treatment plans.

## 4 DISCUSSION

Our findings showed equivalent dose‐volume metrics when comparing the clinical plan and the RapidPlan model‐based plan according to the varying beam energies, MLC types, and disease sites. Besides, the variation of dose indices among RapidPlan models was small. Thus, RapidPlan models have the potential to create consistent treatment plans regardless of these factors and work well for different settings. Now, the Varian treatment machine can generate models for TrueBeam and Clinac iX with the Millennium MLC, for TrueBeam STx with HD, and for Halcyon in this study. To the best of our knowledge, the differences between the clinical plan and the RapidPlan model‐based plan have not yet been compared for these treatment machines. We hope that RapidPlan has the potential to eliminate quality disparities not only regarding interplanner variability but also for generating consistent treatment plans among different treatment machines.

In HNC patients, 6 MV or 6‐MV FFF was used with the test dataset to evaluate the MLC‐type differences in these models. Hong et al. reported that MLC with a finer leaf width (2.5 mm) showed better dosimetric characteristics, providing better dose conformity to the target and reducing spinal cord and peripheral doses in HNC patients; however, no significant difference in dosimetric error was observed according to the MLC leaf width.^25^ Li et al. compared TrueBeam of the Millennium 120 MLC with the Halcyon dual‐layer MLC.^26^ They described that the MLC width may still have an effect on normal tissue doses, although statistical differences were not observed.^26^ Consequently, the effect of MLC width on dosimetric deviations in HNC VMAT plans was small. The dose distributions at PTVs and OARs created by the RapidPlan model in our test dataset were similar to those of the prior study.^26^ In RapidPlan, the dose distribution in the OAR is partitioned into four regions: overlap region between PTV and OAR, in‐field region, MLC transmission region, and out‐of‐field region. These four regions were used to calculate the GED and predict the new DVH. MLC‐type differences affected the dose distribution in the MLC transmission region, although this region was smaller than other regions.^17^ Thus, the effect of the MLC type on dose distribution in the RapidPlan model‐based plan was associated with low‐to‐middle dose distributions, in particular to OARs.

In PK cases, the effect of energy types on the differences between clinical plans and RapidPlan model‐based plans was small. PK evaluated with various energy types demonstrated that the GED in the open‐loop phase could be accurately calculated using different energy types in the closed‐loop phase. The volume ratio between the overlap region and OAR is one index related to the complexity of the treatment plan.^2^ Many OARs in PK, such as the duodenum and stomach, were included in the PTV. Energy selection is important in understanding the behavior of photons for predicting GED. Different energies affect the GED prediction in the overlap region between the target and OAR and in the OAR dose distribution. We also noted that the number of cases with higher individual OAR doses was adequately sparing when using RapidPlan because V_36Gy_ and V_39Gy_ for OARs were reduced compared to the clinical plan. The relationship between PTV and OAR dose varies between RapidPlan versions. Through RapidPlan version 13, the overlap region of GED estimated for OAR was modeled using the average and SD model of the overlap DVH between target and OAR. Alternatively, in the latest version (version 15), the overlap region can be divided into several subregions, one per each model target. Thus, OAR overlap with each model is calculated independently, resulting in more accurate estimates in the OAR region. Due to the new function in RapidPlan, dominance of dose constraints in targeting and OAR is clear; therefore, adequate dose distributions can be constructed.

RC cases involved the thickest body region. Thus, the beam energies employed affected the differences between clinical plans and RapidPlan models. For this region, classical radiotherapy principles recommend higher beam energy for deep‐seated large tumors, but lower energy can reduce the exit dose. However, Ost et al. and Yadav et al. reported negligible differences among various energy types when VMAT was used.^27^, ^28^ VMAT for RC was also reported as a result with respect to RapidPlan.^21^ They clarified that the investigated 10X, 8X, and 6X photons and higher energies provide better normal tissue sparing in RapidPlan.^21^ Our findings suggested that higher beam energies were still advantageous in normal tissue sparing for deep‐seated tumors of large volumes, even if many beam entries were used, such as in the VMAT technique.

According to the manufacturer, the target parameters are needed to determine the fixed objectives because RapidPlan does not estimate DVHs. Huang et al. mentioned that target parameter in RapidPlan models configured for flattened beams cannot optimize un‐flattened beams without adjustments.^21^ However, the authors did not validate to adjust optimization parameters. Castriconi et al. mentioned that the validation step can enhance the robustness of the RapidPlan model to reproduce the plans with the same quality.^6^ We validated the optimization parameter using the validation dataset; thus, the dose distribution in the target was comparable between the flatted and unflatted beams. Moreover, the disease sites were compared to evaluate the effects of the number of affected sites on the RapidPlan models. Our results showed that the number of targets did not affect the target dose, in addition to a reduction in OAR dosage. The RapidPlan algorithm calculates photon behavior and patient geometry regarding the target position. Our results indicate that RapidPlan accurately suggests the GED and predicts the DVH in multiple targets.

Although interplanner variability is considered to have a significant impact on dose distribution, the impact of mechanical constraints due to energy and MLC type is also considered to be site‐specific. Our findings showed that RapidPlan has the potential for creating consistency in treatment planning; therefore, it helps promote multi‐institutional research. The multicenter performance of RapidPlan has been previously reported.^14^, ^15^ These studies indicated that the effects of RapidPlan models, including several different energy and MLC types, were not considered. Furthermore, Kavanaugh et al. and Tol et al. concluded that RapidPlan models can be applied as a patient‐specific quality assurance tool in multi‐institutional clinical trials.^16^, ^29^ In their studies, a multi‐institutional treatment plan was judged based on whether the clinical plan was comparable with the RapidPlan model created by a single institution. Although these studies presented evidence showing that a single‐institutional RapidPlan model can provide patient‐specific quality assurance for the treatment plan in clinical trials, it was beyond the scope of these studies to determine the optimal method for model generation.^16^, ^29^ Our findings may provide evidence that RapidPlan can create consistent, reliable plans for multi‐institutional research when contouring and treatment planning concepts are equivalent. RapidPlan could be beneficial to clinical and, in particular, to prospective research because consistency in contouring and planning concepts is provided by the clinical trial protocol.

The usefulness of RapidPlan for different treatment planning systems was reported by two research groups. Cagni et al. mentioned that RapidPlan created using helical tomotherapy plans was suitable for generating clinically acceptable plans,^30^ and Ueda et al. showed that RapidPlan provided appropriate intermediate doses compared to clinical plans, which were optimized with RayStation.^31^ These findings, in conjunction with ours, indicate that RapidPlan can be suitable for intertechnique, intersystem, and intermachine applications in esophageal cancer. The mechanical performance of RapidPlan was acceptable for clinical use without any major problems.^32^ Presently, the clinical applicability of RapidPlan is limited due to the dedicated Varian system. However, it may be potentially useful in all situations because, compared to manual optimization in VMAT, RapidPlan facilitates irradiation without adding much mechanical load.

A possible limitation of the present work is that a small patient population was used to analyze the effect of machine parameters on outcome variability. The potential improvement of the RapidPlan results requires future work to assess larger patient cohorts. In this study, we used a 10‐patient test dataset to compare the dataset of the same size with all disease sites. Enrolled patients with RC (*N* = 30) were less than other disease sites. Moreover, the 20‐patient dataset was required to build a model in RapidPlan. As a result, the test dataset could only be used for 10 patients. Second, the effects of other Varian treatment machines using RapidPlan, such as Edge, Novalis Tx, VitalBeam, Trilogy, and Unique, were not investigated. However, the accelerator construction in these machines is similar to TrueBeam, TrueBeam STx, and Clinac iX; thus, the results should be comparable with our findings. Third, the small number of objectives was used to evaluate the dosimetric indices. Specifically, for HNC, only two critical structures (spinal cord and parotid grands) were used. Fourth, only specific dosimetric indices and not the overall quality of the plans were assessed.

## 5 CONCLUSION

Dosimetric indices of RapidPlan model‐based plans appear to be comparable to the ones based on clinical plans regardless of energies, MLC types, and disease sites. The results of our study suggest that the RapidPlan model can be used to formulate treatment planning models, independent of the type of treatment machine.

## AUTHOR CONTRIBUTIONS

HH performed the planning study and statistical analysis and drafted the manuscript. HH, MN, NM, RA, KF, and KN conceived the study, participated in its design and coordination, and helped to draft the manuscript. All authors read and approved the final manuscript.

## Supporting information


**Table S1** Dose constraints for head and neck cancer planning.

**Table S2** Dose constraints for pancreatic cancer planning.

**Table S3** Dose constraints for rectal cancer planning.

**Table S4** Objective template as defined in the RapidPlan model for automatic optimization in treating head and neck cancer.

**Table S5** Objective template as defined in the RapidPlan model for automatic optimization in treating pancreatic cancer.

**Table S6** Objective template as defined in the RapidPlan model for automatic optimization in treating rectal cancer.

**Table S7** Characteristics of the examined treatment machines used in this study.

**Table S8** Summary of PTV and OAR volumes in the training and test datasets.
