# Supplementary material for: Reducing variability among treatment machines using knowledge‐based planning for head and neck, pancreatic, and rectal cancer
Source: J Appl Clin Med Phys. 2021 Jun 20;22(7):245–54. doi: 10.1002/acm2.13316 (PMC8292706; doi:10.1002/acm2.13316)
Supplement: Supplementary file 1 — Table S1 Dose constraints for head and neck cancer planning. [file ACM2-22-245-s002.docx]

# Supplementary Tables

**Supplementary Table 1** Dose constraints for head and neck cancer planning

| Structure | Dosimetric parameter | Dose constraint | Unit |
| --- | --- | --- | --- |
| PTV70 | D_50%_ | =7000 | cGy |
|  | D_98%_ | >6510 | cGy |
|  | D_2%_ | <7350 | cGy |
| PTV63 | D_90%_ | =6300 | cGy |
|  | D_50%_ | <6615 | cGy |
| PTV56 | D_90%_ | =5600 | cGy |
|  | D_50%_ | <5880 | cGy |
| Spinal cord | D_max_ | <4500 | cGy |
| Left parotid | V_30Gy_ | <50 | % |
| Right parotid | V_30Gy_ | <50 | % |

Abbreviations: D_max_ = maximum dose; D_mean_ = mean dose; D_xx_% = dose covering xx% volume of a structure’s region; PTVxx = structure delivered xx Gy to the planning target volume; V_yy_Gy = volume receiving yy Gy.
