# Supplementary material for: Reducing variability among treatment machines using knowledge‐based planning for head and neck, pancreatic, and rectal cancer
Source: J Appl Clin Med Phys. 2021 Jun 20;22(7):245–54. doi: 10.1002/acm2.13316 (PMC8292706; doi:10.1002/acm2.13316)
Supplement: Supplementary file 2 — Table S2 Dose constraints for pancreatic cancer planning. [file ACM2-22-245-s008.docx]

**Supplementary Table 2** Dose constraints for pancreatic cancer planning

| Structure | Dosimetric parameter | Dose constraint | Unit |
| --- | --- | --- | --- |
| PTV-PRVs | D_95%_ | =4200 | cGy |
| PTV | D_98%_ | >3600 | cGy |
| GTV-PRV | D_50%_ | =4500 | cGy |
| Spinal cord | D_max_ | <3600 | cGy |
| Stomach | V_42Gy_ | <0.5 | cc |
|  | V_39Gy_ | <1 | cc |
|  | V_36Gy_ | <20 | cc |
| Duodenum | V_42Gy_ | <0.5 | cc |
|  | V_39Gy_ | <1 | cc |
|  | V_36Gy_ | <20 | cc |
| Kidney | V_20Gy_ | <30 | % |
| Liver | D_mean_ | <3000 | cGy |
| Bile duct | D_max_ | <4200 | cGy |

Abbreviation: D_max_ = maximum dose; D_mean_ = mean dose; D_xx%_ = dose covering xx% volume of a structure’s region; GTV = gross tumor volume; PRVs = planning organ at risk volumes for stomach and duodenum; PTV = planning target volume; V_yyGy_ = volume receiving yy Gy.
