# Supplementary material for: Reducing variability among treatment machines using knowledge‐based planning for head and neck, pancreatic, and rectal cancer
Source: J Appl Clin Med Phys. 2021 Jun 20;22(7):245–54. doi: 10.1002/acm2.13316 (PMC8292706; doi:10.1002/acm2.13316)
Supplement: Supplementary file 3 — Table S3 Dose constraints for rectal cancer planning. [file ACM2-22-245-s003.docx]

**Supplementary Table 3** Dose constraints for rectal cancer planning

| Structure | Dosimetric parameter | Dose constraint | Unit |
| --- | --- | --- | --- |
| PTV | D_98%_ | >3600 | cGy |
|  | D_50%_ | >4500 | cGy |
|  | D_2%_ | >5170 | cGy |
| PTV-PRVs | D_98%_ | >3820 | cGy |
|  | D_2%_ | >4950 | cGy |
| Overlap PTV-PRVs | D_98%_ | >3600 | cGy |
|  | D_2%_ | >5170 | cGy |
| PRV small bowel | V_15Gy_ | <120 (if possible) | cc |
|  | V_45Gy_ | <0 (if possible) | cc |
| PRV large bowel | V_15Gy_ | <120 (if possible) | cc |
|  | V_45Gy_ | <0 (if possible) | cc |

Abbreviation: D_xx%_ = dose covering xx% volume of region of structure; Overlap = overlap structure between PTV and PRVs; PRVs = planning organ at risk volumes for small bowel and large bowel; PTV = planning target volume; V_yyGy_ = volume receiving yy Gy.
