# Supplementary material for: Reducing variability among treatment machines using knowledge‐based planning for head and neck, pancreatic, and rectal cancer
Source: J Appl Clin Med Phys. 2021 Jun 20;22(7):245–54. doi: 10.1002/acm2.13316 (PMC8292706; doi:10.1002/acm2.13316)
Supplement: Supplementary file 4 — Table S4 Objective template as defined in the RapidPlan model for automatic optimization in treating head and neck cancer. [file ACM2-22-245-s001.docx]

**Supplementary Table 4** Objective template as defined in the RapidPlan model for automatic optimization in treating head and neck cancer

| Organs | Objectives | Volume [%] | Dose [cGy] | Priority |
| --- | --- | --- | --- | --- |
| CTV56 | Upper | 0.1 | 5880 | Generated |
|  | Upper | 3 | 5740 | Generated |
|  | Lower | 99.9 | 5530 | 190 |
| CTV63 | Upper | 0.1 | 6580 | Generated |
|  | Upper | 3 | 6440 | 150 |
|  | Lower | 99.9 | 6230 | 200 |
| CTV70 | Upper | 0 | 7280 | Generated |
|  | Upper | 1 | 7210 | Generated |
|  | Lower | 99.9 | 7035 | 160 |
| PTV56-CTV56 | Upper | 0.1 | 5880 | Generated |
|  | Upper | 3 | 5810 | Generated |
|  | Lower | 99.9 | 5390 | 180 |
| PTV63-CTV63 | Upper | 0.1 | 6580 | Generated |
|  | Upper | 3 | 6440 | 150 |
|  | Lower | 99.9 | 6230 | 200 |
| PTV70-CTV70 | Upper | 0.1 | 7070 | Generated |
|  | Lower | 99.9 | 6930 | 160 |
| Spinal cord | Upper | 0 | 4200 | Generated |
|  | Line (Preferring OAR) | Generated | Generated | Generated |
| Brain stem | Upper | 0 | 4700 | Generated |
|  | Line (Preferring OAR) | Generated | Generated | Generated |
| Lungs | Line (Preferring target) | Generated | Generated | Generated |
| Mandible | Upper | 0.1 | 6900 | Generated |
|  | Line (Preferring target) | Generated | Generated | Generated |
| Ring | Upper | Generated | 6300 | Generated |
|  | Upper | Generated | 5000 | Generated |
| Opt neck | Upper | 0 | 3400 | 200 |
|  | Line (Preferring target) | Generated | Generated | Generated |
| Opt left parotid | Upper | 60 | 1000 | 120 |
|  | Upper | 40 | 1500 | 150 |
|  | Upper | 20 | 2000 | Generated |
|  | Line (Preferring OAR) | Generated | Generated | Generated |
| Opt right parotid | Upper | 60 | 1000 | 120 |
|  | Upper | 40 | 1500 | 150 |
|  | Upper | 20 | 2000 | Generated |
|  | Line (Preferring OAR) | Generated | Generated | Generated |
| PRV spinal cord | Line (Preferring target) | Generated | Generated | Generated |
| PRV brain stem | Line (Preferring target) | Generated | Generated | Generated |

Abbreviations: CTV = clinical target volume; OAR = organ at risk; Opt left/right parotid = optimization structure subtracting 4 mm from the expanded PTV for the parotid structure; Opt neck = optimization structure to adjust the dose distribution on the dorsal side of the PTV; PRV = planning organ at risk volume; PTV = planning target volume; Ring = the structure subtract 5 mm extended around PTV from 20 mm extended around PTV.
