# Supplementary material for: Reducing variability among treatment machines using knowledge‐based planning for head and neck, pancreatic, and rectal cancer
Source: J Appl Clin Med Phys. 2021 Jun 20;22(7):245–54. doi: 10.1002/acm2.13316 (PMC8292706; doi:10.1002/acm2.13316)
Supplement: Supplementary file 5 — Table S5 Objective template as defined in the RapidPlan model for automatic optimization in treating pancreatic cancer. [file ACM2-22-245-s007.docx]

**Supplementary Table 5** Objective template as defined in the RapidPlan model for automatic optimization in treating pancreatic cancer

| Organs | Objectives | Volume [%] | Dose [cGy] | Priority |
| --- | --- | --- | --- | --- |
| GTV | Upper | 0 | 4550 | 200 |
|  | Lower | 98 | 4500 | 120 |
| Overlap PTV-PRVs | Upper | 3 | 4250 | 70 |
|  | Upper | 30 | 3950 | 60 |
|  | Upper | 50 | 3850 | 60 |
|  | Lower | 99 | 3650 | 100 |
| PTV | Upper | 0 | 4400 | 160 |
|  | Upper | 2 | 4350 | 60 |
|  | Lower | 98 | 4300 | 150 |
|  | Lower | 100 | 4250 | 160 |
| Bile duct | Upper | 0 | 4100 | 180 |
|  | Line (Preferring target) | Generated | Generated | Generated |
| Large bowel | Upper | 0 | 3900 | 140 |
|  | Line (Preferring target) | Generated | Generated | Generated |
| Small bowel | Upper | 0 | 3800 | 170 |
|  | Line (Preferring target) | Generated | Generated | Generated |
| Duodenum | Upper | 0 | 3800 | 200 |
|  | Upper | 0 | 4000 | 100 |
|  | Line (Preferring OAR) | Generated | Generated | Generated |
| PRV duodenum | Upper | 0 | 4000 | 200 |
|  | Upper | 1 | 3800 | 100 |
|  | Line (Preferring OAR) | Generated | Generated | Generated |
| Left kidney | Line (Preferring target) | Generated | Generated | Generated |
| Right kidney | Line (Preferring target) | Generated | Generated | Generated |
| Liver | Line (Preferring target) | Generated | Generated | Generated |
| Spinal cord | Line (Preferring OAR) | Generated | Generated | Generated |
| PRV Spinal cord | Upper | 0 | 3500 | Generated |
|  | Line (Preferring OAR) | Generated | Generated | Generated |
| Stomach | Upper | 0 | 4000 | 200 |
|  | Upper | 1 | 3800 | 100 |
|  | Line (Preferring OAR) | Generated | Generated | Generated |
| PRV stomach | Upper | 0 | 4000 | 160 |
|  | Upper | 1 | 3800 | 160 |
|  | Line (Preferring OAR) | Generated | Generated | Generated |

Abbreviations: GTV = gross tumor volume; OAR = organ at risk; PRV = planning organ at risk volume; PTV = planning target volume; PRV duodenum = 5 mm extended around duodenum; PRV spinal cord = 5 mm extended around spinal cord; PRV stomach =10 mm extended around stomach.
