# Supplementary material for: Reducing variability among treatment machines using knowledge‐based planning for head and neck, pancreatic, and rectal cancer
Source: J Appl Clin Med Phys. 2021 Jun 20;22(7):245–54. doi: 10.1002/acm2.13316 (PMC8292706; doi:10.1002/acm2.13316)
Supplement: Supplementary file 6 — Table S6 Objective template as defined in the RapidPlan model for automatic optimization in treating rectal cancer. [file ACM2-22-245-s004.docx]

**Supplementary Table 6** Objective template as defined in the RapidPlan model for automatic optimization in treating rectal cancer

| Organs | Objectives | Volume [%] | Dose [cGy] | Priority |
| --- | --- | --- | --- | --- |
| PTV | Upper | 0 | 4635 | 200 |
|  | Lower | 100 | 4401 | 300 |
| Overlap PTV-Bowel | Upper | 0.1 | 4401 | 250 |
|  | Lower | 99.9 | 4149 | 300 |
| PTV-overlap structure | Upper | 0 | 4635 | 250 |
|  | Lower | 99 | 4450.5 | 250 |
|  | Lower | 99.9 | 4351.5 | 250 |
| Bladder | Line (Preferring target) | Generated | Generated | Generated |
| Large Bowel | Upper | 0 | 4200 | 200 |
|  | Line (Preferring OAR) | Generated | Generated | Generated |
| Small Bowel | Upper | 0 | 4200 | 200 |
|  | Line (Preferring OAR) | Generated | Generated | Generated |

Abbreviation: PTV = planning target volume; OAR = organ at risk
