# Supplementary material for: Reducing variability among treatment machines using knowledge‐based planning for head and neck, pancreatic, and rectal cancer
Source: J Appl Clin Med Phys. 2021 Jun 20;22(7):245–54. doi: 10.1002/acm2.13316 (PMC8292706; doi:10.1002/acm2.13316)
Supplement: Supplementary file 7 — Table S7 Characteristics of the examined treatment machines used in this study. [file ACM2-22-245-s005.docx]

**Supplementary Table 7** Characteristics of the examined treatment machines used in this study

| Treatment machine | Energy | MLC | Max. field size |
| --- | --- | --- | --- |
| TrueBeam STx | 6 MV, 10 MV, 15 MV,  6-MV FFF, 10-MV FFF | 2.5 mm, 5 mm (HD120) | 22 × 22 cm |
| TrueBeam | 6 MV | 5 mm, 10 mm (Millennium 120) | 40 × 40 cm |
| Clinac iX | 6 MV, 15 MV | 5 mm, 10 mm (Millennium 120) | 40 × 40 cm |
| Halcyon | 6-MV FFF | 5 mm (dual-layer MLC) | 28 × 28 cm |

Abbreviations: FFF = flattening filter free; HD = high definition; MLC = multi-leaf collimator.
