# Supplementary material for: Reducing variability among treatment machines using knowledge‐based planning for head and neck, pancreatic, and rectal cancer
Source: J Appl Clin Med Phys. 2021 Jun 20;22(7):245–54. doi: 10.1002/acm2.13316 (PMC8292706; doi:10.1002/acm2.13316)
Supplement: Supplementary file 8 — Table S8 Summary of PTV and OAR volumes in the training and test datasets. [file ACM2-22-245-s006.docx]

**Supplementary Table 8** Summary of PTV and OAR volumes in the training and test datasets

| Volume [cc] | PTV | | | | | | OAR | | | | | |
| --- | --- | --- | --- | --- | --- | --- | --- | --- | --- | --- | --- | --- |
| Head and Neck | PTV70 | | PTV63 | | PTV56 | | Spinal cord | | Left parotid | | Right parotid | |
|  | Training | Test | Training | Test | Training | Test | Training | Test | Training | Test | Training | Test |
|  | 135.9±14.8 | 151.7±20.2 | 359.2±21.8 | 406.1±44.1 | 249.3±23.8 | 205.6±58.3 | 32.7±1.2 | 40.1±3.4 | 26.0±1.8 | 26.2±2.2 | 26.5±1.5 | 24.3±2.1 |
|  |  |  |  |  |  |  |  |  |  |  |  |  |
| Pancreas | PTV | |  |  |  |  | Spinal cord | | Stomach | | Duodenum | |
|  | Training | Test |  |  |  |  | Training | Test | Training | Test | Training | Test |
|  | 176.4±6.1 | 160.3±9.0 |  |  |  |  | 43.6±1.4 | 36.4±4.6 | 186.5±6.9 | 199.0±13.0 | **56.7±2.1** | **68.1±4.1** |
|  |  |  |  |  |  |  |  |  |  |  |  |  |
| Rectum | PTV | |  |  |  |  | Large bowel | | Small bowel | |  |  |
|  | Training | Test |  |  |  |  | Training | Test | Training | Test |  |  |
|  | 887.1±37.6 | 1013.6±107.2 |  |  |  |  | 273.9±42.6 | 193.9±68.5 | 350.1±43.2 | 330.8±61.2 |  |  |

Abbreviations: PTV = planning target volume; OAR = organ at risk.

Statistical analysis: Data are presented as the mean ± standard deviation (cc). Values in bold represent a statistically significant difference (p<0.05, student t-test). The comparison between the training and test datasets took into account the results of the normality and equal variance tests.
